# Supplementary material for: COVID-19 and mental health deterioration by ethnicity and gender in the UK
Source: PLoS One. 2021 Jan 6;16(1):e0244419. doi: 10.1371/journal.pone.0244419 (PMC7787387; doi:10.1371/journal.pone.0244419)
Supplement: S1 Appendix — (DOCX) [file pone.0244419.s001.docx]

**S1 Appendix. GHQ-12 Questionnaire.**

The GHQ module contains the following questions for the GHQ-12 Questionnaire:

- scghqa [GHQ: concentration] The next questions are about how you have been feeling over the last few weeks. Have you recently been able to concentrate on whatever you're doing?

1. Better than usual 2. Same as usual 3. Less than usual 4. Much less than usual

- scghqb [GHQ: loss of sleep] Have you recently lost much sleep over worry?

1. Not at all 2. No more than usual 3. Rather more than usual 4. Much more than usual

- scghqc [GHQ: playing a useful role] Have you recently felt that you were playing a useful part in things?

1. More so than usual 2. Same as usual 3. Less so than usual 4. Much less than usual

- scghqd [GHQ: capable of making decisions] Have you recently felt capable of making decisions about things?

1. More so than usual 2. Same as usual 3. Less so than usual 4. Much less capable

- scghqe [GHQ: constantly under strain] Have you recently felt constantly under strain?

1. Not at all 2. No more than usual 3. Rather more than usual 4. Much more than usual

- scghqf [GHQ: problem overcoming difficulties] Have you recently felt you couldn't overcome your difficulties?

1. Not at all 2. No more than usual 3. Rather more than usual 4. Much more than usual

- scghqg [GHQ: enjoy day-to-day activities] Have you recently been able to enjoy your normal day-to-day activities?

1. More so than usual 2. Same as usual 3. Less so than usual 4. Much less than usual

- scghqh [GHQ: ability to face problems] Have you recently been able to face up to problems?

1. More so than usual 2. Same as usual 3. Less able than usual 4. Much less able

- scghqi [GHQ: unhappy or depressed] Have you recently been feeling unhappy or depressed?

1. Not at all 2. No more than usual 3. Rather more than usual 4. Much more than usual

- scghqj [GHQ: losing confidence] Have you recently been losing confidence in yourself?

1. Not at all 2. No more than usual 3. Rather more than usual 4. Much more than usual

- scghqk [GHQ: believe worthless] Have you recently been thinking of yourself as a worthless person?

1. Not at all 2. No more than usual 3. Rather more than usual 4. Much more than usual

- scghql [GHQ: general happiness] Have you recently been feeling reasonably happy, all things considered?

1. More so than usual 2. About the same as usual 3. Less so than usual 4. Much less than usual

The GHQ-12 range goes from 0-36. This range is obtained by subtracting 1 to the values given in each question. Thus, the values in each question are re-coded from 1-4 to 0-3.
