# Supplementary material for: COVID-19 and mental health deterioration by ethnicity and gender in the UK
Source: PLoS One. 2021 Jan 6;16(1):e0244419. doi: 10.1371/journal.pone.0244419 (PMC7787387; doi:10.1371/journal.pone.0244419)
Supplement: S2 Appendix — (DOCX) [file pone.0244419.s002.docx]

**S2 Appendix. Definition of variables.**

**Outcome (dependent) variables:**

The outcome (dependent) variables are based on:

- The **subjective wellbeing (GHQ): Likert** (*scghq1_dv*). *scghq1_dv* “converts valid answers to 12 questions of the General Health Questionnaire (GHQ) to a single scale by recoding so that the scale for individual variables runs from 0 to 3 instead of 1 to 4, and then summing, giving a scale running from 0 (the least distressed) to 36 (the most distressed).”

<https://www.understandingsociety.ac.uk/documentation/mainstage/dataset-documentation/variable/scghq1_dv>

- The **subjective wellbeing (GHQ): Caseness** (*scghq2_dv*). *scghq2_dv* “converts valid answers to 12 questions of the General Health Questionnaire (GHQ) to a single scale by recoding 1 and 2 values on individual variables to 0, and 3 and 4 values to 1, and then summing, giving a scale running from 0 (the least distressed) to 12 (the most distressed).”

<https://www.understandingsociety.ac.uk/documentation/mainstage/dataset-documentation/variable/scghq2_dv>

**Explanatory (independent) variables:**

- The ethnic group indicators are created using *racel_dv*, which “uses information collected in interviews with adults and includes information collected in previous interviews and from other members of the household.” We define BAME as all people other than White British (*racel_dv==1*): <https://www.devon.gov.uk/equality/communities/diversity/guide/race>. In other words, BAME people include all people with *racel_dv!=1*, including Other White defined as (*racel_dv==2 | racel_dv==3 | racel_dv==4*). <https://www.understandingsociety.ac.uk/documentation/mainstage/dataset-documentation/variable/racel_dv>
- Gender indicator is based on *sex==sex_dv*. <https://www.understandingsociety.ac.uk/documentation/covid-19/dataset-documentation/variable/sex>

<https://www.understandingsociety.ac.uk/documentation/covid-19/dataset-documentation/variable/sex_dv>

- The age group indicators in April 2020 (<25, 25-34, 35-44, 45-54, 55-64, and 65+) are based on *age* (age in April 2020). <https://www.understandingsociety.ac.uk/documentation/covid-19/dataset-documentation/variable/age>
- The face-to-face interview indicator in 2017-2019 (=1 if face-to-face, = 0 else) is based on *indmode*.

<https://www.understandingsociety.ac.uk/documentation/mainstage/dataset-documentation/variable/indmode>

- The month of interview indicators in 2017-2019 is based on *intdatm_dv*.

<https://www.understandingsociety.ac.uk/documentation/mainstage/dataset-documentation/variable/intdatm_dv>

- Household size in April 2020 (approx.) is based on the sum of the household composition indicators: *hhcompa* (Household composition – Aged 0-4), *hhcompb* (Household composition – Aged 5-15), *hhcompc* (Household composition – Aged 16-18), *hhcompd* (Household composition – Aged 19-69) and *hhcompe* (Household composition – Aged 70 or older).
- <https://www.understandingsociety.ac.uk/documentation/covid-19/dataset-documentation?covid_19_variables=composition>
- The indicator for living with a partner in April 2020 is based on *couple*. <https://www.understandingsociety.ac.uk/documentation/covid-19/dataset-documentation/variable/couple>
- The location indicators (England (excluding London), London, Northern Ireland, Wales, Scotland) are created using *gor_dv*. <https://www.understandingsociety.ac.uk/documentation/mainstage/dataset-documentation/variable/gor_dv>
- The educational group indicators in 2017-2019 are created using *qfhigh_dv*: BA or higher (*qfhigh_dv <=2*), diploma or equivalent (*qfhigh_dv >2 & qfhigh_dv <=6*), A Level or equivalent (*qfhigh_dv >6 & qfhigh_dv <=12*), GCSE or equivalent (*qfhigh_dv >12 & qfhigh_dv <=16*), none of the above (*qfhigh_dv >16 & qfhigh_dv !=.*) <https://www.understandingsociety.ac.uk/documentation/mainstage/dataset-documentation/variable/qfhigh_dv>
- The employment status in 2017-2019 is based on *jbstat*: self-employed (*jbstat==1*), employed (*jbstat==2*) unemployed (*jbstat==3*), retired (*jbstat==4*), family care or home (*jbstat==6*), student (*jbstat==7*), disabled (*jbstat==8*), other (*jbstat==5 | jbstat==9 | jbstat==10 | jbstat==11 | jbstat==12*).

<https://www.understandingsociety.ac.uk/documentation/mainstage/dataset-documentation/variable/jbstat>

- The personal income measure in 2017-2019 is the monthly total net personal income - no deductions (*fimnnet_dv*) divided by 1,000.

<https://www.understandingsociety.ac.uk/documentation/mainstage/dataset-documentation/variable/fimnnet_dv>

- The health condition indicator is based on the 22 binary indicators (at least one of the health conditions): *hcond_cv1 hcond_cv2 hcond_cv3 hcond_cv4 hcond_cv5 hcond_cv6 hcond_cv7 hcond_cv8 hcond_cv11 hcond_cv21 hcond_cv10 hcond_cv12 hcond_cv13 hcond_cv14 hcond_cv15 hcond_cv16 hcond_cv22 hcond_cv19 hcond_cv23 hcond_cv24 hcond_cv27 hcond_cv18*.

<https://www.understandingsociety.ac.uk/documentation/covid-19/dataset-documentation?covid_19_variables=hcond_cv>

**Survey weights:**

- We use sample weights based on *indinui_xw*: <https://www.understandingsociety.ac.uk/documentation/mainstage/dataset-documentation/variable/indinui_xw>.
