# Supplementary material for: COVID-19 and mental health deterioration by ethnicity and gender in the UK
Source: PLoS One. 2021 Jan 6;16(1):e0244419. doi: 10.1371/journal.pone.0244419 (PMC7787387; doi:10.1371/journal.pone.0244419)
Supplement: S1 Table — (DOCX) [file pone.0244419.s003.docx]

**S1 Table. Table 4 with reported coefficients on control variables.**

|  | Difference | | Standardised diff. | | Difference | |
| --- | --- | --- | --- | --- | --- | --- |
|  | GHQ-12 score | | GHQ-12 score | | GHQ “caseness” score | |
|  | (1) | (2) | (3) | (4) | (5) | (6) |
| Female | 0.995*** | 1.035*** | 0.166*** | 0.173*** | 0.092*** | 0.100*** |
|  | (0.130) | (0.143) | (0.022) | (0.024) | (0.012) | (0.013) |
| BAME | 0.843*** | 0.921** | 0.141*** | 0.154** | 0.053** | 0.064** |
|  | (0.314) | (0.360) | (0.052) | (0.060) | (0.027) | (0.031) |
| Female × BAME | -0.754 | -0.820 | -0.126 | -0.137 | -0.049 | -0.062 |
|  | (0.475) | (0.500) | (0.079) | (0.084) | (0.040) | (0.043) |
| <25 |  | 2.312*** |  | 0.386*** |  | 0.174*** |
|  |  | (0.589) |  | (0.098) |  | (0.049) |
| 25-34 |  | 1.588*** |  | 0.265*** |  | 0.112*** |
|  |  | (0.336) |  | (0.056) |  | (0.031) |
| 35-44 |  | 0.804** |  | 0.134** |  | 0.073*** |
|  |  | (0.318) |  | (0.053) |  | (0.028) |
| 45-54 |  | -0.008 |  | -0.001 |  | 0.018 |
|  |  | (0.258) |  | (0.043) |  | (0.024) |
| 65+ |  | 0.242 |  | 0.040 |  | 0.018 |
|  |  | (0.216) |  | (0.036) |  | (0.021) |
| Living with a partner |  | -0.159 |  | -0.026 |  | 0.015 |
|  |  | (0.188) |  | (0.031) |  | (0.017) |
| January |  | -0.127 |  | -0.021 |  | -0.053* |
|  |  | (0.322) |  | (0.054) |  | (0.029) |
| February |  | -0.230 |  | -0.038 |  | -0.015 |
|  |  | (0.344) |  | (0.057) |  | (0.031) |
| March |  | 0.017 |  | 0.003 |  | -0.034 |
|  |  | (0.338) |  | (0.056) |  | (0.029) |
| May |  | -0.002 |  | -0.000 |  | -0.039 |
|  |  | (0.327) |  | (0.055) |  | (0.029) |
| June |  | 0.162 |  | 0.027 |  | 0.002 |
|  |  | (0.336) |  | (0.056) |  | (0.031) |
| July |  | 0.052 |  | 0.009 |  | -0.004 |
|  |  | (0.337) |  | (0.056) |  | (0.029) |
| August |  | 0.019 |  | 0.003 |  | -0.019 |
|  |  | (0.309) |  | (0.052) |  | (0.028) |
| September |  | 0.279 |  | 0.047 |  | -0.009 |
|  |  | (0.356) |  | (0.059) |  | (0.030) |
| October |  | -0.013 |  | -0.002 |  | -0.010 |
|  |  | (0.322) |  | (0.054) |  | (0.028) |
| November |  | -0.309 |  | -0.052 |  | -0.051* |
|  |  | (0.327) |  | (0.055) |  | (0.030) |
| December |  | -0.484 |  | -0.081 |  | -0.036 |
|  |  | (0.338) |  | (0.056) |  | (0.030) |
| Face-to-face |  | 0.212 |  | 0.035 |  | 0.003 |
|  |  | (0.147) |  | (0.025) |  | (0.013) |
| Household size |  | -0.020 |  | -0.003 |  | -0.008 |
|  |  | (0.066) |  | (0.011) |  | (0.006) |
| London |  | 0.454 |  | 0.076 |  | 0.024 |
|  |  | (0.292) |  | (0.049) |  | (0.026) |
| Wales |  | 0.412 |  | 0.069 |  | 0.013 |
|  |  | (0.314) |  | (0.053) |  | (0.028) |
| Scotland |  | 0.265 |  | 0.044 |  | 0.020 |
|  |  | (0.261) |  | (0.044) |  | (0.023) |
| Northern Ireland |  | -0.044 |  | -0.007 |  | 0.034 |
|  |  | (0.495) |  | (0.083) |  | (0.039) |
| BA or higher |  | 0.189 |  | 0.032 |  | 0.039** |
|  |  | (0.200) |  | (0.033) |  | (0.018) |
| Diploma or equivalent |  | -0.045 |  | -0.007 |  | 0.019 |
|  |  | (0.251) |  | (0.042) |  | (0.022) |
| A Level or equivalent |  | -0.045 |  | -0.007 |  | 0.015 |
|  |  | (0.269) |  | (0.045) |  | (0.024) |
| GCSE or equivalent |  | -0.118 |  | -0.020 |  | 0.001 |
|  |  | (0.201) |  | (0.034) |  | (0.018) |
| Self-employed |  | 0.647** |  | 0.108** |  | 0.044* |
|  |  | (0.271) |  | (0.045) |  | (0.025) |
| Unemployed |  | -2.142*** |  | -0.358*** |  | -0.208*** |
|  |  | (0.524) |  | (0.087) |  | (0.047) |
| Retired |  | 0.729*** |  | 0.122*** |  | 0.038* |
|  |  | (0.219) |  | (0.037) |  | (0.021) |
| Family care or home |  | -0.525 |  | -0.088 |  | -0.083** |
|  |  | (0.474) |  | (0.079) |  | (0.038) |
| Student |  | -0.798 |  | -0.133 |  | -0.126** |
|  |  | (0.682) |  | (0.114) |  | (0.049) |
| Disabled |  | -2.595*** |  | -0.433*** |  | -0.226*** |
|  |  | (0.520) |  | (0.087) |  | (0.042) |
| Other |  | 0.643 |  | 0.107 |  | 0.083 |
|  |  | (0.725) |  | (0.121) |  | (0.063) |
| Net personal income (£1K) |  | 0.031 |  | 0.005 |  | 0.002 |
|  |  | (0.044) |  | (0.007) |  | (0.004) |
| Health conditions |  | -0.053 |  | -0.009 |  | 0.000 |
|  |  | (0.147) |  | (0.025) |  | (0.013) |
| Observations | 12,516 | 10,920 | 12,516 | 10,920 | 12,516 | 10,920 |
| R-squared | 0.007 | 0.029 | 0.007 | 0.029 | 0.007 | 0.024 |
