# Supplementary material for: COVID-19 and mental health deterioration by ethnicity and gender in the UK
Source: PLoS One. 2021 Jan 6;16(1):e0244419. doi: 10.1371/journal.pone.0244419 (PMC7787387; doi:10.1371/journal.pone.0244419)
Supplement: S2 Table — (DOCX) [file pone.0244419.s004.docx]

**S2 Table. Table 6 with reported coefficients on control variables.**

|  | Difference | | Standardised diff. | | Difference | |
| --- | --- | --- | --- | --- | --- | --- |
|  | GHQ-12 score | | GHQ-12 score | | GHQ “caseness” score | |
|  | (1) | (2) | (3) | (4) | (5) | (6) |
| Female | 0.995*** | 1.035*** | 0.166*** | 0.173*** | 0.092*** | 0.100*** |
|  | (0.130) | (0.143) | (0.022) | (0.024) | (0.012) | (0.013) |
| BIP | 1.859*** | 1.547*** | 0.310*** | 0.258*** | 0.100*** | 0.087** |
|  | (0.393) | (0.425) | (0.066) | (0.071) | (0.038) | (0.040) |
| Non-BIP | 0.488 | 0.677 | 0.081 | 0.113 | 0.037 | 0.056 |
|  | (0.390) | (0.452) | (0.065) | (0.076) | (0.033) | (0.038) |
| Female × BIP | -1.426** | -1.496** | -0.238** | -0.250** | -0.027 | -0.028 |
|  | (0.624) | (0.657) | (0.104) | (0.110) | (0.061) | (0.062) |
| Female × Non-BIP | -0.490 | -0.564 | -0.082 | -0.094 | -0.052 | -0.068 |
|  | (0.578) | (0.619) | (0.097) | (0.103) | (0.048) | (0.052) |
| <25 |  | 2.308*** |  | 0.385*** |  | 0.174*** |
|  |  | (0.588) |  | (0.098) |  | (0.049) |
| 25-34 |  | 1.586*** |  | 0.265*** |  | 0.112*** |
|  |  | (0.336) |  | (0.056) |  | (0.031) |
| 35-44 |  | 0.806** |  | 0.135** |  | 0.074*** |
|  |  | (0.318) |  | (0.053) |  | (0.028) |
| 45-54 |  | -0.006 |  | -0.001 |  | 0.019 |
|  |  | (0.258) |  | (0.043) |  | (0.024) |
| 65+ |  | 0.242 |  | 0.040 |  | 0.018 |
|  |  | (0.216) |  | (0.036) |  | (0.021) |
| Living with a partner |  | -0.162 |  | -0.027 |  | 0.015 |
|  |  | (0.188) |  | (0.031) |  | (0.017) |
| January |  | -0.125 |  | -0.021 |  | -0.053* |
|  |  | (0.322) |  | (0.054) |  | (0.029) |
| February |  | -0.228 |  | -0.038 |  | -0.015 |
|  |  | (0.344) |  | (0.058) |  | (0.031) |
| March |  | 0.019 |  | 0.003 |  | -0.034 |
|  |  | (0.338) |  | (0.056) |  | (0.029) |
| May |  | -0.002 |  | -0.000 |  | -0.039 |
|  |  | (0.327) |  | (0.055) |  | (0.029) |
| June |  | 0.162 |  | 0.027 |  | 0.002 |
|  |  | (0.336) |  | (0.056) |  | (0.031) |
| July |  | 0.058 |  | 0.010 |  | -0.004 |
|  |  | (0.337) |  | (0.056) |  | (0.029) |
| August |  | 0.021 |  | 0.004 |  | -0.019 |
|  |  | (0.309) |  | (0.052) |  | (0.028) |
| September |  | 0.281 |  | 0.047 |  | -0.008 |
|  |  | (0.356) |  | (0.059) |  | (0.030) |
| October |  | -0.009 |  | -0.001 |  | -0.009 |
|  |  | (0.322) |  | (0.054) |  | (0.028) |
| November |  | -0.304 |  | -0.051 |  | -0.050* |
|  |  | (0.327) |  | (0.055) |  | (0.030) |
| December |  | -0.484 |  | -0.081 |  | -0.036 |
|  |  | (0.338) |  | (0.056) |  | (0.030) |
| Face-to-face |  | 0.209 |  | 0.035 |  | 0.002 |
|  |  | (0.147) |  | (0.025) |  | (0.013) |
| Household size |  | -0.022 |  | -0.004 |  | -0.008 |
|  |  | (0.066) |  | (0.011) |  | (0.006) |
| London |  | 0.452 |  | 0.076 |  | 0.023 |
|  |  | (0.292) |  | (0.049) |  | (0.026) |
| Wales |  | 0.417 |  | 0.070 |  | 0.013 |
|  |  | (0.315) |  | (0.053) |  | (0.029) |
| Scotland |  | 0.274 |  | 0.046 |  | 0.021 |
|  |  | (0.261) |  | (0.044) |  | (0.023) |
| Northern Ireland |  | -0.014 |  | -0.002 |  | 0.038 |
|  |  | (0.498) |  | (0.083) |  | (0.039) |
| BA or higher |  | 0.188 |  | 0.031 |  | 0.039** |
|  |  | (0.201) |  | (0.033) |  | (0.018) |
| Diploma or equivalent |  | -0.047 |  | -0.008 |  | 0.020 |
|  |  | (0.251) |  | (0.042) |  | (0.022) |
| A Level or equivalent |  | -0.046 |  | -0.008 |  | 0.015 |
|  |  | (0.269) |  | (0.045) |  | (0.024) |
| GCSE or equivalent |  | -0.120 |  | -0.020 |  | 0.001 |
|  |  | (0.201) |  | (0.034) |  | (0.018) |
| Self-employed |  | 0.650** |  | 0.109** |  | 0.044* |
|  |  | (0.271) |  | (0.045) |  | (0.024) |
| Unemployed |  | -2.129*** |  | -0.355*** |  | -0.209*** |
|  |  | (0.524) |  | (0.088) |  | (0.047) |
| Retired |  | 0.728*** |  | 0.121*** |  | 0.037* |
|  |  | (0.219) |  | (0.037) |  | (0.021) |
| Family care or home |  | -0.520 |  | -0.087 |  | -0.084** |
|  |  | (0.474) |  | (0.079) |  | (0.038) |
| Student |  | -0.800 |  | -0.134 |  | -0.126** |
|  |  | (0.682) |  | (0.114) |  | (0.050) |
| Disabled |  | -2.593*** |  | -0.433*** |  | -0.225*** |
|  |  | (0.520) |  | (0.087) |  | (0.042) |
| Other |  | 0.647 |  | 0.108 |  | 0.083 |
|  |  | (0.725) |  | (0.121) |  | (0.063) |
| Net personal income (£1K) |  | 0.032 |  | 0.005 |  | 0.002 |
|  |  | (0.044) |  | (0.007) |  | (0.004) |
| Health conditions |  | -0.052 |  | -0.009 |  | 0.000 |
|  |  | (0.147) |  | (0.025) |  | (0.013) |
| Observations | 12,516 | 10,920 | 12,516 | 10,920 | 12,516 | 10,920 |
| R-squared | 0.007 | 0.029 | 0.007 | 0.029 | 0.007 | 0.024 |
